# Spatiotemporal Dynamics of Scrub Typhus Transmission in Mainland China, 2006-2014

**DOI:** 10.1371/journal.pntd.0004875

**Published:** 2016-08-01

**Authors:** Yi-Cheng Wu, Quan Qian, Ricardo J. Soares Magalhaes, Zhi-Hai Han, Wen-Biao Hu, Ubydul Haque, Thomas A. Weppelmann, Yong Wang, Yun-Xi Liu, Xin-Lou Li, Hai-Long Sun, Yan-Song Sun, Archie C. A. Clements, Shen-Long Li, Wen-Yi Zhang

**Affiliations:** 1 Institute of Disease Control and Prevention, Academy of Military Medical Science, Beijing, People’s Republic of China; 2 Spatial Epidemiology Laboratory, School of Veterinary Science, The University of Queensland, Brisbane, Australia; 3 Child Health Research Center, The University of Queensland, Brisbane, Australia; 4 Navy General Hospital of PLA, Beijing, People’s Republic of China; 5 School of Public Health and Social Work, Queensland University of Technology, Brisbane, Australia; 6 Emerging Pathogens Institute, University of Florida, Gainesville, Florida, United States of America; 7 Department of Geography, University of Florida, Gainesville, Florida, United States of America; 8 Department of Environmental and Global Health, College of Public Health and Health Professions, University of Florida, Gainesville, Florida, United States of America; 9 Chinese People’s Liberation Army General Hospital, Beijing, People’s Republic of China; 10 517 Military Hospital, Kelan, Shanxi, People’s Republic of China; 11 Academy of Military Medical Science, Beijing, People’s Republic of China; 12 Research School of Population Health, The Australian National University, Canberra, Australian Capital Territory, Australia; Institut Pasteur, FRANCE

## Abstract

**Background:**

Scrub typhus is endemic in the Asia-Pacific region including China, and the number of reported cases has increased dramatically in the past decade. However, the spatial-temporal dynamics and the potential risk factors in transmission of scrub typhus in mainland China have yet to be characterized.

**Objective:**

This study aims to explore the spatiotemporal dynamics of reported scrub typhus cases in mainland China between January 2006 and December 2014, to detect the location of high risk spatiotemporal clusters of scrub typhus cases, and identify the potential risk factors affecting the re-emergence of the disease.

**Method:**

Monthly cases of scrub typhus reported at the county level between 2006 and 2014 were obtained from the Chinese Center for Diseases Control and Prevention. Time-series analyses, spatiotemporal cluster analyses, and spatial scan statistics were used to explore the characteristics of the scrub typhus incidence. To explore the association between scrub typhus incidence and environmental variables panel Poisson regression analysis was conducted.

**Results:**

During the time period between 2006 and 2014 a total of 54,558 scrub typhus cases were reported in mainland China, which grew exponentially. The majority of cases were reported each year between July and November, with peak incidence during October every year. The spatiotemporal dynamics of scrub typhus varied over the study period with high-risk clusters identified in southwest, southern, and middle-eastern part of China. Scrub typhus incidence was positively correlated with the percentage of shrub and meteorological variables including temperature and precipitation.

**Conclusions:**

The results of this study demonstrate areas in China that could be targeted with public health interventions to mitigate the growing threat of scrub typhus in the country.

## Author Summary

Scrub typhus is a vector-borne disease carried by the chigger mite and is endemic in the Asia-Pacific region. Now scrub typhus causes a considerable burden on public health and the economy in China. We explored the spatiotemporal dynamics of scrub typhus cases in China between January 2006 and December 2014, and explored the potential risk factors affecting the spatial distribution of the disease. The majority of cases were reported between July and November, with peak incidence during October every year. Several high-risk clusters were identified in southwest, southern, and middle-east China. Scrub typhus incidence was positively correlated with the percentage of shrub, and temporal variation in temperature and precipitation in China.

## Introduction

Scrub typhus, also known as tsutsugamushi disease, is endemic in the so-called “tsutsugamushi triangle” area that includes Pakistan and Afghanistan in the west, far-eastern Russia and Japan in the north, and northern Australia in the south [1]. The causative bacterium of this disease, *Orientia tsutsugamushi* (*O*. *tsutsugamushi*), is spread to humans bitten by infected species of trombiculid mites [2, 3]. The clinical presentation of scrub typhus is characterized by high fever and rash or typical eschar at the location of the bite, which can progress to multiple organ failure and even death in some cases [4–6]. It is estimated that over one billion people are currently living in at-risk areas and approximately one million cases occur around the world annually [2, 7]. In recent years, there has been a drastic increase in both the frequency and geographic distribution of scrub typhus cases, which could signal the re-emergence of this neglected tropical disease [8–11].

The first reported case of a human infected with scrub typhus in China was identified in the southern province of Guangdong in 1948 [12]. Until the 1980s, scrub typhus cases primarily occurred in the regions south of Yangtze River with established natural foci including Zhejiang in the east and Yunnan in the west part of China [13, 14]. However, with rapid societal development, changing environment, climate change, population movement, better recognition by health care professionals and ever-improving detection techniques, both sporadic cases and disease outbreaks began to be identified in the northern provinces of Shandong, Jiangsu, Tianjin and Beijing, as well as the emergence of new natural foci in the past three decades [13, 15–17]. Currently, the disease is widespread in most of the provinces in mainland China, where the incidence has increased rapidly in recent years. Despite the recent resurgence of illness, scrub typhus remains a neglected tropical disease that is able to simultaneously impact tourism and military activities in China [9, 18], with the potential to cause a significant burden on both public health and economy.

In the past decades, spatiotemporal analysis techniques have been widely applied in the surveillance of infectious disease and outbreak investigations [19–21]. Previous studies have identified clusters of reported cases of scrub typhus in different provinces of China and reported that the geographic distribution of the disease varied by year [22, 23]. Some studies have revealed that environmental variables were important drivers in the transmission of scrub typhus [7, 24]. However, there are few studies that examine both the spatiotemporal dynamics and potential risk factors in scrub typhus transmission across China. Thus, the objectives of this study were to describe the temporal trends in scrub typhus incidence, to detect spatiotemporal clusters of scrub typhus cases at the county level, and to identify the physical environmental variables associated with scrub typhus incidence, which would be helpful for the health administration officers and public health workers to the implementation of effective intervention measures targeted toward high-risk areas and populations.

## Materials and Methods

### Ethics statement

This study was approved by the Ethics Committee of Beijing Institute of Disease Control and Prevention. All the data analyzed in this study were de-identified to protect patient confidentiality.

### Data collection and management

In China, scrub typhus is a vector-borne notifiable disease; attending physicians are required by law to report to the China Center for Disease Control and Prevention through the China Information System for Disease Control and Prevention (CISDCP). Scrub typhus case reports include basic demographic and clinical data including gender, age, occupation, residential address, date of onset of symptoms, laboratory diagnosis, and clinical outcome for each case. Data from January 2006 through December 2014 were obtained from CISDCP. All scrub typhus cases were confirmed according to the diagnostic criteria issued by the Ministry of Health of the People’s Republic of China. The criteria for a confirmed case of scrub typhus include epidemiological exposure histories (travelling to an endemic area and contact with chiggers or rodents within 3 weeks before the onset of illness), clinical manifestations (such as high fever, lymphadenopathy, skin rash and eschar or ulcers), and also at least one of the laboratory diagnosis: a 4-fold or greater rise in serum IgG antibody titers between acute and convalescent sera by using indirect immunofluorescence antibody assay (IFA), or detection of *O*. *tsutsugamushi* by polymerase chain reaction (PCR) in clinical specimens, or isolation of *O*. *tsutsugamushi* from clinical specimens [24–26].

Demographic data for each county was obtained from the National Bureau of Statistics of China. Environmental and meteorological data from 2006 to 2014 were collected. Land cover variables such as the percentage coverage of cropland, forest, shrub, grassland, built-up land and water bodies were collected from the data on land cover 2005 and 2009 released by European space agency (http://www.esa.int). Meteorological variables including temperature, relative humidity and precipitation were obtained from the Chinese Academy of Meteorological Sciences (www.cams.cma.gov.cn).

In order to perform spatial analysis, the data set of cases was aggregated at the county level as the spatial unit for analysis. In mainland China, there are 31 provinces (or municipalities) comprised of 2,922 counties, with population sizes ranging from 7,123 to 5,044,430, with geographic areas ranging in size from 5.4 to 197,346 square kilometres. All cases were geocoded and matched to the county-level administrative boundaries using the ArcGIS software (version 9.3, ESRI, Redlands, CA).

### Temporal analysis of scrub typhus incidence

The cases of scrub typhus reported at the county-level were aggregated to provide a national data set of monthly cases for time-series analyses. The monthly incidence as well as the cumulative number of cases was tabulated for visualization, along with the graphical assessment of the cumulative annual cases with various trends including linear, polynomial, and exponential growth curves using Excel (Microsoft, Redmond, WA). For the assessment of the seasonal trend in scrub typhus incidence, both the annual and long-term trends were assessed. The average monthly incidence for every calendar month (January to December) was compared to the average incidence in January (the lowest monthly incidence and beginning of each year) using a categorical Poisson regression model to generate incident rate ratios (IRR) and 95% confidence intervals (CI) for the IRR. Temporal autocorrelation between the monthly reported cases of scrub typhus and seasonal trend in incidence was assessed using time lags between 0 and 60 months. Seasonal trends were classified by maximum autocorrelations of 12 months and minimum autocorrelations observed every 6 months, that also demonstrated a sinusoidal oscillation with respect to time.

### Spatiotemporal cluster analysis

Local Indicators of Spatial Association (LISA) were used to assess the spatial pattern of scrub typhus incidence at the county level during the study period. LISA was used to identify significant hotspots (High-High), coldspots (Low-Low), and outliers (High-Low and Low-High) by calculating local Moran’s I index between a given county and the neighbouring values in the surrounding counties [27]. The significance level of clusters was determined using a Z score generated by comparison of the Local Moran’s I statistic for the average incidence in each county. A high positive Z score indicated that the surroundings had spatial clusters (High-High: high-value spatial clusters or Low-Low: low-value spatial clusters) and a low negative Z score indicated the presence of spatial outliers (High-Low: high values surrounded with low values or Low-High: Low values surrounded with high values) [27]. Kulldorff’s space-time scan statistic (SaTScan software, version 9.1.1) was used to explore the location of high-risk space-time clusters. The space-time scan statistic was defined by a cylindrical window with a circular (or elliptic) geographic base and with height corresponding to time [28]. The base was defined exactly as for the purely spatial scan statistic, while the height reflected the time period of the potential clusters [28]. In this study, circular scan windows were selected and fit discrete Poisson models. The maximum spatial cluster size was set to 5% of the population at risk in the spatial window and a maximum temporal cluster size of 10% of the study period in the temporal window. Likelihood ratio tests were evaluated to determine the significance of identified clusters and P-values were obtained through Monte Carlo simulation after 999 replications. The null hypothesis of a spatiotemporally random distribution was rejected when the P-value was < 0.05.

### Association between yearly scrub typhus incidence and environmental factors

We conducted panel Poisson regression analysis to examine the association between yearly scrub typhus incidence and potential environment risk factors. An autocorrelation term was included to account for spatial and temporal dependency in scrub typhus incidence. The autocorrelation term was calculated using the minimum distance from each county-center to the nearest cluster. After aggregating the yearly incidence into a panel dataset, the association between incidence and environmental factors was examined, with IRR and their corresponding 95% confidence intervals and p values estimated using maximum likelihood methods. The temporal analysis and the panel Poisson regression analysis were conducted in STATA software (Stata Crop Lp, College Station, TX, USA).

## Results

### Temporal analysis of scrub typhus incidence

**Fig 1:**
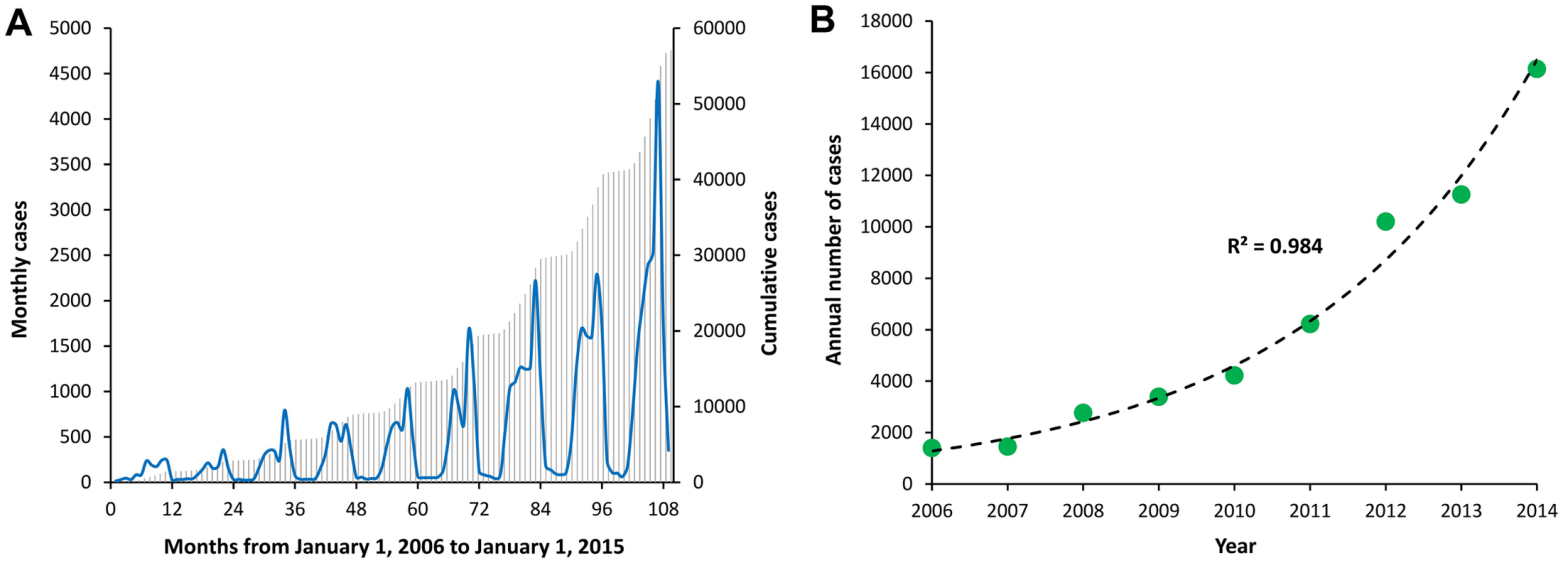
Temporal distribution of scrub typhus cases from 2006 to 2014 in mainland China. The temporal distributions of reported scrub typhus cases in mainland China are presented as the monthly number of cases (blue line) and cumulative cases (gray spikes) in panel A and the total number of cases reported per year (blue dots) fit with an exponential growth function (black line) in panel B.

**Fig 2:**
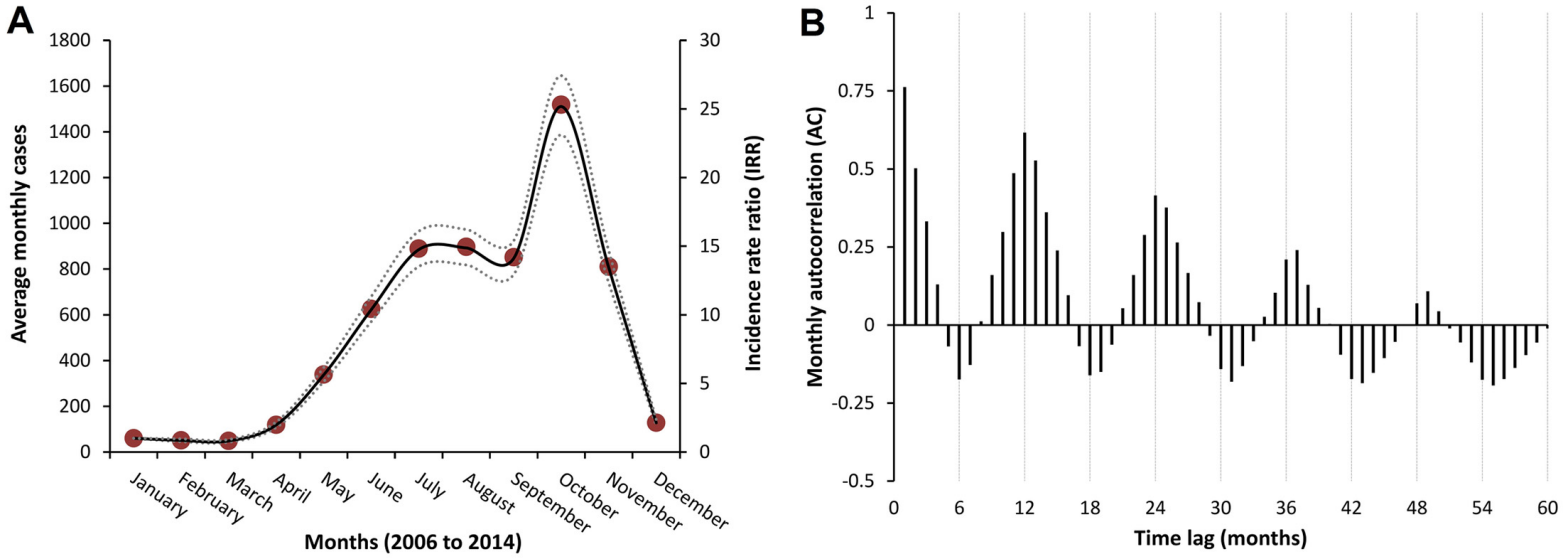
Seasonal patterns in reported scrub typhus cases from 2006 to 2014 in mainland China. The seasonal patterns of reported scrub typhus cases in mainland China are presented as the average number of cases reported by month (blue dots) and the incidence rate ratios (IRR) with 95% confidence intervals for the IRR (dashed and dotted lines) derived from a categorical Poisson regression models for the number of reported cases per month, with all months compared to the number of cases reported in January of each year in panel A. The seasonal patterns as determined by monthly autocorrelation between the number of reported cases for each month is presented using time lags between 0 and 60 months in panel B.

A total of 54,558 scrub typhus cases were reported from 1,031 counties during the period between 2006 and 2014. The monthly variations in the number of scrub typhus cases presented in Fig 1A suggested a seasonal relationship, whereas the rapid increase in the total number of annual cases in Fig 1B was explained by an exponential growth function (R^2^ = 0.98). Scrub typhus cases occurred throughout the year, however began to increase dramatically in April through September, before reaching a peak in October and returning to low, constant levels of transmission in the winter months of December through March. The average numbers of reported cases are presented by month in Fig 2A, along with the incidence rate ratios comparing each month to January of each year. It was estimated that the month of October, which consistently had the largest number of reported cases, had an incidence of scrub typhus that was approximately 25 times higher than the winter months of January, February, or March (P <0.001); IRR = 25.2; 95% CI: (13.1, 27.4). Given the seasonal patterns observed in Fig 1A, the autocorrelation of the monthly scrub typhus cases was compared using a time-series analysis. The pattern of autocorrelation in Fig 2B not only demonstrated that the monthly reported incidence in one month was significantly correlated with the previous months’ incidence, but also exhibited maximum correlations in every 12 months, minimum correlations in every 6 months, and followed a sinusoidal pattern of oscillation with an increasing correlation between 2006 and 2014. Though aggregated for the temporal analyses, the annual incidence rate of scrub typhus was highly variable at the county level, which ranged from zero reported cases to 66.21 cases per 100,000 residents.

### Spatiotemporal cluster analysis

**Fig 3:**
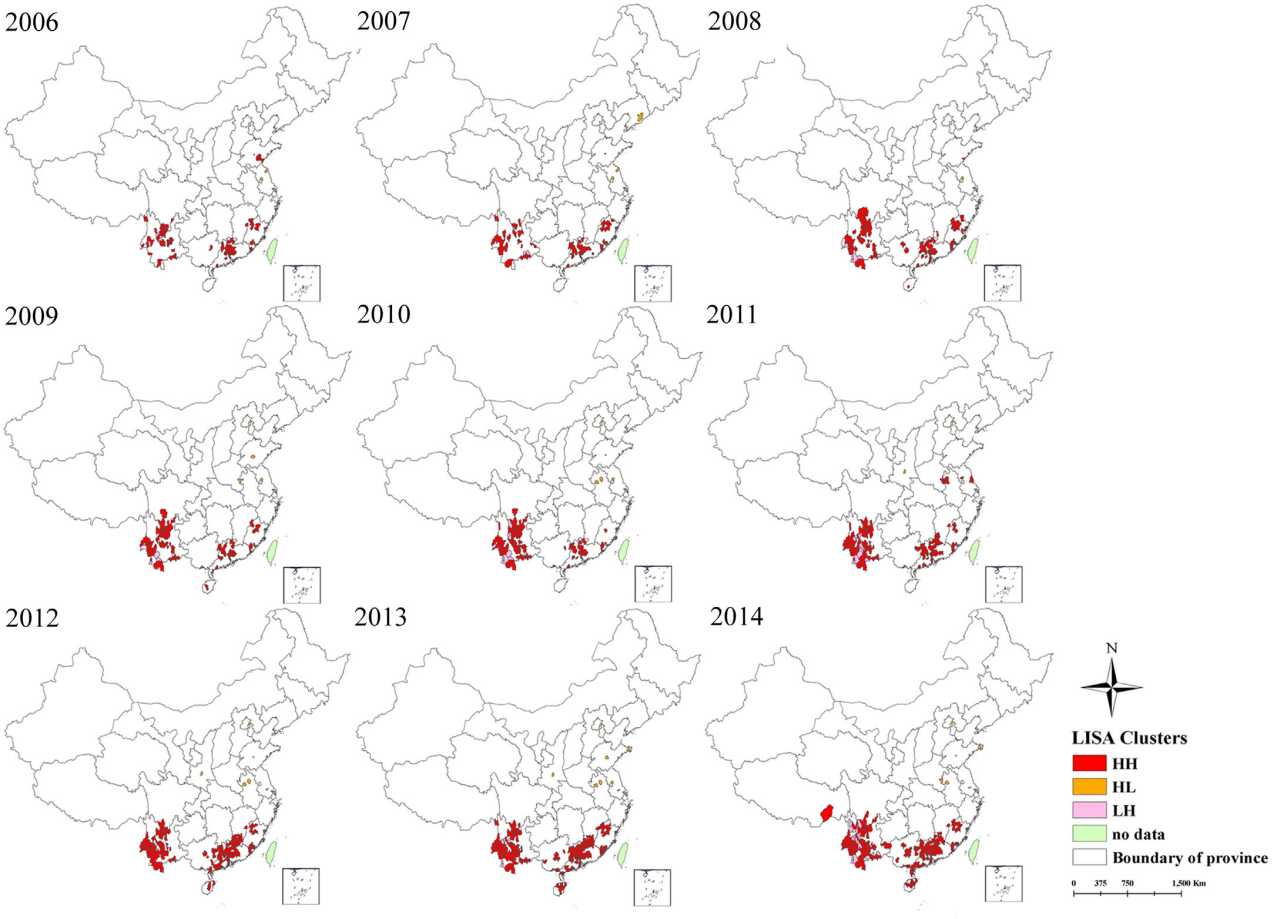
Yearly Local Indicators of Spatial Association (LISA) cluster maps for scrub typhus incidence in mainland China, 2006–2014. LISA spatial cluster map shows the center of the cluster in color. H-H indicates a statistically significant cluster of high scrub typhus incidence values; H-L represents high scrub typhus incidence values surrounded with low incidence values; L-H represents low scrub typhus incidence values surrounded with high incidence values.

Spatial analysis of scrub typhus incidence at the county level demonstrated the spatial autocorrelation was positive, indicating clustering of reported cases during the study period. The values of Moran’s *I* ranged from 0.02 to 0.08 with all P values < 0.05 (Table 1), indicating the presence of clusters of scrub typhus incidence in each year. The hotspots (High-High) and outliers of scrub typhus transmission in mainland China were identified through LISA analysis. Hotspots were primarily distributed in the southern and southwestern provinces of China, however variation of the location was observed. In 2006, the hotspots were distributed sporadically in Yunnan, Guangdong and Fujian. Later hotspots in those provinces expanded to include larger geographic areas over the next eight years (Fig 3). Hotspots also occurred and expanded in Guangxi and Hainan province between 2008 and 2014, with a short appearance in the northern provinces of Anhui and Jiangsu in 2011. High-Low outliers were sporadically distributed in the middle-eastern provinces of China including Shandong, Jiangsu and Anhui, while Low-High outliers were mainly concentrated in Yunnan province (Fig 3). During the study period, the proportion of counties and populations within High-High clusters increased from 1.51% to 6.19% and 1.44% to 5.95% respectively. Additionally, hotspot counties were responsible for between 48.96% of all reported cases in 2006 to 67.57% in 2013 (Table 2).

**Table 1:** Yearly spatiotemporal autocorrelation analysis on scrub typhus incidence in mainland China, 2006–2014.

Year	Moran's *I*	Z-Score	P-value
2006	0.02	9.59	<0.05
2007	0.03	18.11	<0.05
2008	0.05	27.33	<0.05
2009	0.05	25.19	<0.05
2010	0.05	27.02	<0.05
2011	0.06	30.44	<0.05
2012	0.06	36.24	<0.05
2013	0.07	38.05	<0.05
2014	0.08	42.43	<0.05

**Table 2:** Descriptive statistics of scrub typhus spatial clusters detected by Local Indicators of Spatial Association analysis in mainland China, 2006–2014.

	Incidence* (1/100000)	% Cases	%Counties	% Population	% Area
2006					
HH	3.23	48.96	1.51	1.44	0.80
HL	10.43	9.62	0.03	0.09	0.02
LH	0.00	0.00	0.00	0.00	0.00
2007					
HH	2.31	61.08	2.91	2.68	1.87
HL	3.17	5.01	0.10	0.16	0.09
LH	0.00	0.00	0.07	0.06	0.05
2008					
HH	3.36	63.64	3.83	3.75	2.75
HL	6.58	0.96	0.03	0.03	0.01
LH	0.00	0.00	0.31	0.18	0.26
2009					
HH	5.72	65.82	3.25	2.82	2.45
HL	3.94	5.82	0.17	0.36	0.07
LH	0.00	0.00	0.21	0.11	0.18
2010					
HH	6.69	59.78	3.25	2.77	2.53
HL	7.95	7.45	0.17	0.29	0.07
LH	0.00	0.00	0.31	0.19	0.37
2011					
HH	6.86	64.80	4.31	4.30	3.02
HL	7.27	1.35	0.07	0.08	0.02
LH	0.00	0.00	0.31	0.20	0.40
2012					
HH	8.11	64.67	5.13	5.37	3.68
HL	11.98	7.22	0.24	0.41	0.09
LH	0.00	0.00	0.03	0.02	0.02
2013					
HH	9.95	67.57	5.99	5.73	3.99
HL	11.46	6.95	0.31	0.51	0.13
LH	0.00	0.00	0.21	0.11	0.13
2014					
HH	12.51	61.18	6.19	5.95	4.67
HL	20.40	7.59	0.24	0.45	0.09
LH	0.14	0.04	0.62	0.38	0.61

Incidence*: annual average incidence, calculated using yearly counts of scrub typhus cases as a numerator and population size in the middle of each year as a denominator; HH: High-High, a statistically significant cluster of high scrub typhus incidence values; HL: High-Low, high scrub typhus incidence values surrounded with low scrub typhus incidence values; LH: Low-High, low scrub typhus incidence values surrounded with high scrub typhus incidence values.

**Fig 4:**
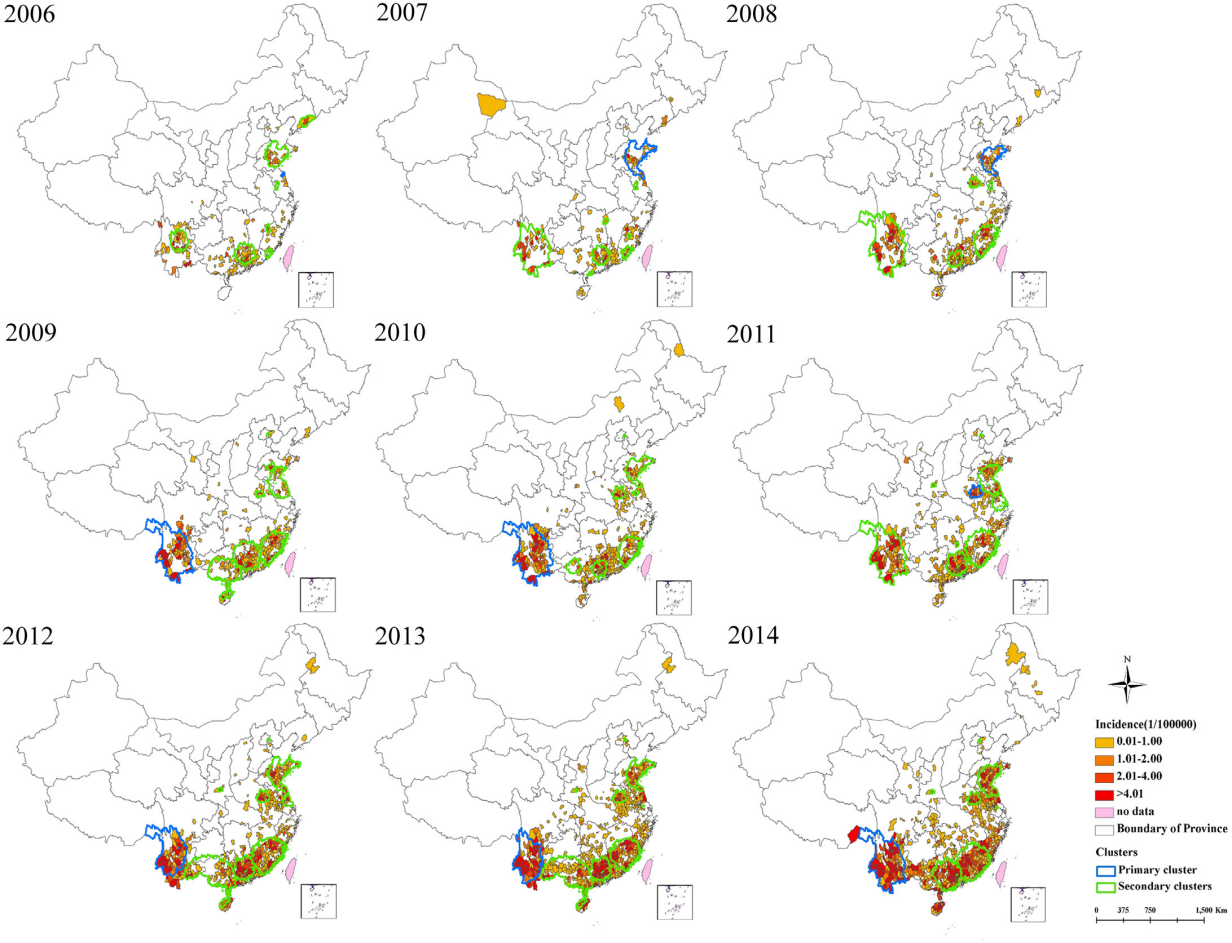
Yearly spatiotemporal clusters overlay with annual incidence of scrub typhus in mainland China, 2006–2014. Yearly spatiotemporal clusters were detected using a circular scan window with the maximum spatial size of 5% of the population at risk and a maximum temporal size of 10% of the study period.

Fig 4 shows the distribution of annual average scrub typhus incidence and the location of spatial clusters identified by using Kulldorff’s space-time scan statistic for each year from 2006 to 2014. As can be seen, both the number of counties with increased scrub typhus incidence expanded persistently between 2006 and 2014, which resulted in the formation of a large, continuous geographic area of scrub typhus incidence in southern mainland China. The primary cluster of scrub typhus cases was originally located in Shandong and Jiangsu province, after which the area expanded between 2006 and 2008. Since that time, the primary cluster of increased scrub typhus incidence shifted to southwest, except for 2011, where the primary cluster was identified in the northwestern region that included Anhui province. Secondary clusters of scrub typhus cases were also identified in southern and southeastern China as well as in Shaanxi and Beijing, with five to eight clusters identified every year.

**Fig 5:**
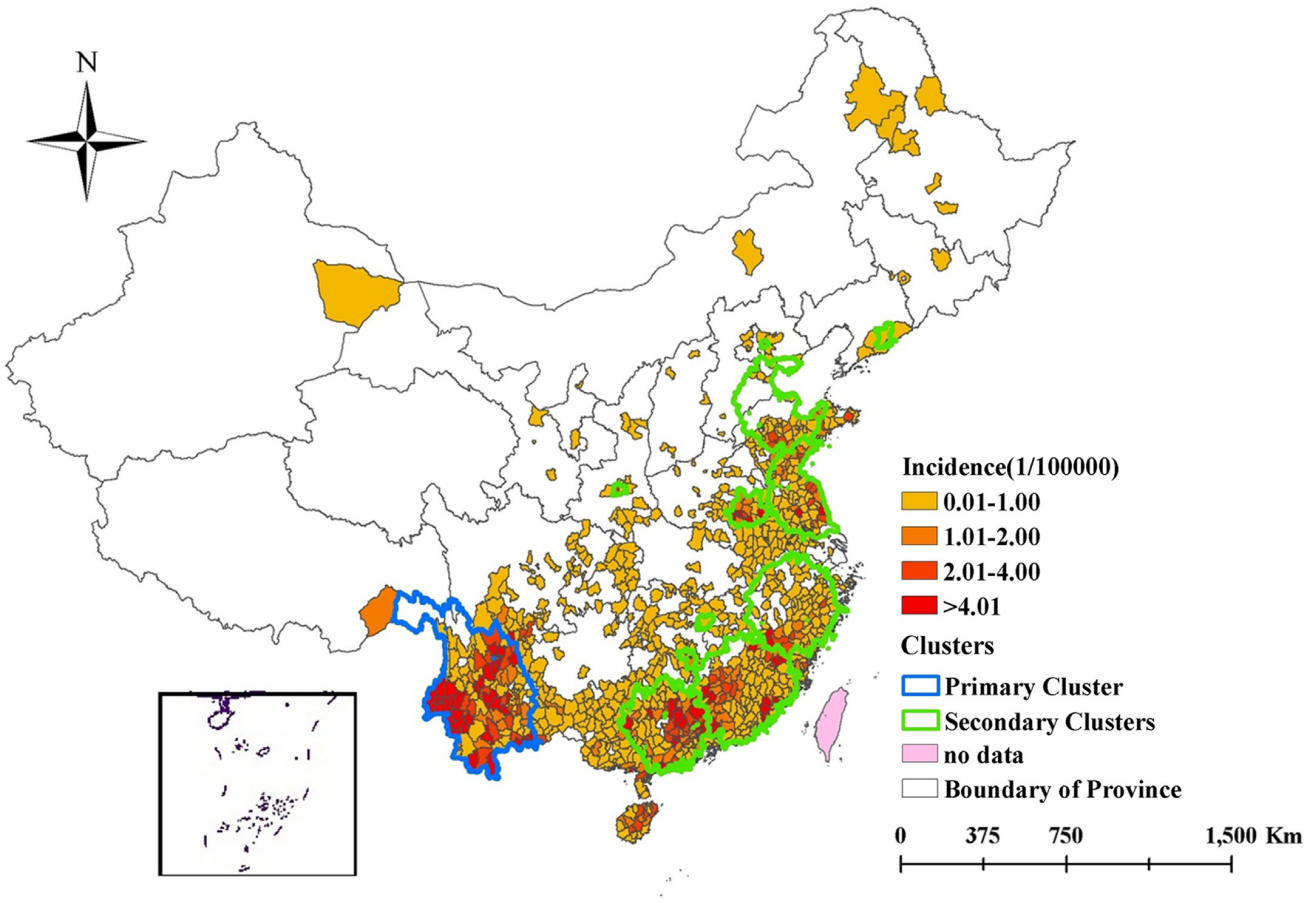
Spatiotemporal clusters overlay with annual average incidence of scrub typhus across the period of 2006–2014 in mainland China.

Additionally, spatiotemporal clusters across the entire study period between 2006 and 2014 were identified by using Kulldorff’s spatiotemporal scan statistic (Fig 5). The primary cluster was located in southwest China, including 103 counties in Yunnan, 11 counties in Sichuan, and even a county in Tibet, with a radius of 491.64 km. The time frame of the primary cluster was from July to October in 2014, which coincided with the largest annual scrub typhus outbreak identified by the time-series analyses and carried a RR of 64.88 and log likelihood ratio (LLR) of 10,460 (Table 3). Most importantly, the primary cluster accounted for only 2.63% of the total population, but included 29.28% of the total cases during that time (Table 4). In addition, there were eleven significant secondary clusters identified, also primarily located in southern and middle-eastern China, with the RR and LLR ranging from 2.94 to 800.71 and 32 to 7433, respectively (Table 3).

**Table 3:** Spatiotemporal clusters of scrub typhus detected using Kulldorff’s space-time scan statistic in mainland China*, 2006–2014.

Clusters	Longitude	Latitude	Radius(Km)	Time Frame	No. Counties	No. Obs	No. Exp	LLR	RR
1※	97.81	24.04	491.64	2014/7-2014/10	115	3313	54.31	10460.19	64.88
2#	111.39	23.58	191.43	2014/5-2014/11	101	3562	176.06	7433.65	21.58
3	116.15	33.16	76.71	2014/10-2014/11	17	1219	12.10	4429.29	103.02
4	120.02	34.09	213.36	2014/10-2014/11	85	1593	45.33	4144.77	36.17
5	116.13	25.13	228.98	2014/5-2014/11	132	1821	178.48	2612.10	10.52
6	118.08	37.76	200.83	2014/10	116	406	25.63	742.51	15.95
7	117.14	40.21	0.00^▲^	2014/10	1	130	0.16	739.12	800.71
8	108.58	34.01	27.82	2012/10-2012/11	2	70	1.16	218.16	60.38
9	118.81	29.01	207.78	2014/6-2014/10	117	320	109.45	133.17	2.94
10	112.63	26.76	40.31	2009/7	6	37	1.19	91.39	31.13
11	124.05	40.58	40.68	2006/10-2006/11	2	18	0.59	44.16	30.59
12	113.71	28.23	0.00	2007/9-2007/10	1	17	1.01	32.00	16.83

*Significant clusters with P<0.01;

^※^1: Primary cluster;

^#^2–12: Secondary clusters;

^▲^: Only 1 county was include in the cluster;

No. Counties: number of counties within clusters; No. Obs: number of observed cases; No. Exp: number of expected cases; LLR: log likelihood ratio; RR: relative risk of the cluster compared with the rest of the country.

**Table 4:** Scrub typhus incidence rate, proportion of population and cases in spatiotemporal clusters detected using Kulldorff’s space-time scan statistic in mainland China, 2006–2014.

Clusters	Time Frame	Incidence* (1/100,000)	% Population	% Case
1※	2014/7-2014/10	9.56	2.63	29.28
2#	2014/5-2014/11	5.61	4.81	23.29
3	2014/10-2014/11	6.92	1.34	19.88
4	2014/10-2014/11	2.51	4.81	25.98
5	2014/5-2014/11	2.81	4.91	11.91
6	2014/10	0.63	4.88	9.18
7	2014/10	24.47	0.04	2.94
8	2012/10-2012/11	4.62	0.11	2.07
9	2014/6-2014/10	0.57	4.29	2.49
10	2009/7	1.21	0.23	5.86
11	2006/10-2006/11	2.34	0.06	3.67
12	2007/9-2007/10	1.28	0.10	3.21

^※^1: Primary cluster;

^#^2–12: Secondary clusters;

Incidence*: Scrub typhus incidence during the clustering time.

### Association between scrub typhus incidence and environmental factors

The results of panel Poisson regression analysis revealed that scrub typhus incidence was positively correlated with the percentage of forest (IRR = 1.17; 95% CI: 1.15, 1.10) and shrub (IRR = 7.52; 95% CI: 7.18, 7.87) as well as temperature (IRR = 1.06; 95% CI: 1.05, 1.07) and precipitation (IRR = 1.01; 95% CI: 1.00, 1.01). Our results also indicate that lower incidence of scrub typhus is associated with the percentage of cropland (IRR = 0.61; 95% CI: 0.60, 0.62), grassland (IRR = 0.23; 95% CI: 0.19, 0.28), built-up land (IRR = 0.64; 95% CI: 0.61, 0.67), water bodies (IRR = 0.25; 95% CI: 0.23, 0.27), and relative humidity (IRR = 0.90; 95% CI: 0.90, 0.91). Except for percentage of forest, which was positively correlated in the univariate analysis but negatively correlated in the multivariate analysis (Table 5), the IRR were similar, with the largest differences observed with the percentage of shrub (IRR: 7.52 vs 1.29) and the IRR for temperature (IRR: 1.06 vs 1.35) in the multivariate analysis.

**Table 5:** The association between Scrub typhus and potential factors by panel Poisson regression analysis.

Variables(Unit)	Univariate analysis		Multivariate analysis	
	Crude IRR (95%CI)	*p* value	Adjusted IRR (95%CI)	*p* value
Percentage of cropland (10%)	0.61(0.60,0.62)	< 0.000	0.65(0.55,0.76)	< 0.000
Percentage of forest (10%)	1.17(1.15,1.20)	< 0.000	0.62(0.53,0.73)	< 0.000
Percentage of grassland (10%)	0.23(0.19,0.28)	< 0.000	0.48(0.36,0.63)	< 0.000
Percentage of shrub (10%)	7.52(7.18,7.87)	< 0.000	1.29(1.09,1.53)	< 0.005
Percentage of built-up land (10%)	0.64(0.61,0.67)	< 0.000	0.46(0.39,0.52)	< 0.000
Percentage of water body (10%)	0.25(0.23,0.27)	< 0.000	0.33(0.28,0.40)	< 0.000
Temperature (1 degree Celsius)	1.06(1.05,1.07)	< 0.000	1.35(1.33,1.37)	< 0.000
Relative humidity (1%)	0.90(0.90,0.91)	< 0.000	0.89(0.89,0.90)	< 0.000
Precipitation (100mm)	1.01(1.00,1.01)	< 0.000	1.01(1.00,1.02)	< 0.000
Autoregressive term (10km)	0.90(0.89,0.91)	< 0.000	0.88(0.87,0.89)	< 0.000

## Discussion

The results of our study indicate that the spatiotemporal transmission of scrub typhus has increased exponentially between 2006 and 2014 and spread throughout much of mainland China. LISA and spatial scan statistics analyses identified significant clusters with respect to both space and time that indicated outbreaks of scrub typhus were primarily located in southwestern and southern China. Given the ability of spatiotemporal analyses based on geographic information systems to assist in the identification of counties with the highest risk of contracting scrub typhus, we suggest that these methods could have further application in both future disease surveillance and planning of mitigation strategies.

In a previous study, we identified the most significant cluster of scrub typhus in the southeastern provinces of Guangdong, Fujian, Jiangxi, and Guangxi [25]. In the current study, the counties at the highest risk were located in the southwestern provinces of Yunnan and Sichuan and accounted for nearly a quarter of total cases during the 2014 outbreak. More importantly, by analyzing annual spatiotemporal clustering, the transmission dynamics appeared to shift between middle-east, southeast, and southwest China. Therefore, we suggest that each of these three high-risk regions be considered for the implementation of targeted interventions such as environmental management, controlling and killing rodent and mites, strengthening personal protection. Given that there was a low incidence of scrub typhus in 2007 in north eastern Xinjiang Uygur Autonomous Region contiguous to Gansu province, we suggest that more investigations be performed to determine if novel cases are as yet unreported in those outlying provinces. Notably, high-high spots detected by LISA analysis were primarily concentrated in southern China and rarely identified in the middle-eastern regions. The high-low outliers that were identified in this region, suggest that scrub typhus incidence in this region was concentrated in a few counties, which could indicate that natural foci of scrub typhus are still forming in this region as they expand into northern China. In addition, our study identified clusters in provinces such as Beijing, Shaanxi, and Anhui, which further confirmed disease outbreaks in these provinces reported by other studies [14, 29, 30]. Thus, our findings will further assist health authorities and public health practitioners through the identification of established foci in southern China as well as the documentation of the emergence of new foci in the north.

The increasing number of reported cases and geographic expansion of scrub typhus in China is partly due to increasing quality of the surveillance system and availability of detection facilities as the increasing investment of health resources. Moreover, environment change and human activities could be important factors contributed to this increasing trend [31, 32]. In this study, our findings demonstrated the percentage of shrub, temperature and precipitation were risk factors associated to the spatiotemporal heterogeneity of scrub typhus notifications in China. A possible explanation is that temperature, precipitation, and shrub may affect the population dynamics and activity levels of chigger mites [7, 33]. Previous studies also suggested the migration of infested rodents or chiggers may have led to the formation new natural foci in provinces of Shandong, Henan, and Beijing since the meteorological and vegetation cover conditions are similar in these areas [34]. Additionally, socio-economic factors could have also served as important drivers for the transmission of scrub typhus in recent years. For instance, the urbanization and change of land use may contribute to the spread of scrub typhus into urban areas by providing suitable habitats such as clearings, grasslands, and riverbanks for vectors and small rodents [35]. Presently, we only explored the association between environmental variables (land cover, weather) and the incidence of scrub typhus. In future, a more well-coordinated and interdisciplinary approach is imperative and urgently needed to explore the relative effects of environmental and socio-economic factors on scrub typhus transmission in mainland China.

While this study brings the important new knowledge on the epidemiology of scrub typhus in China, there are also some limitations. Since the case data were obtained from a passive surveillance system, the reporting system might miss some cases due to lack of diagnostic facilities and/or misdiagnosed due to the co-occurrence of other febrile diseases such as leptospirosis, typhoid fever, or hemorrhagic fever in the absence of the characteristic eschar [36, 37]. Additionally, in our analysis we chose to use a circular scan window in space-time scan statistics. While the circular scan has been documented to perform better at detecting larger clusters compared to the elliptic window scan, it may also include insignificant zones and has been shown to have reduced performance when used with irregular shapes [38, 39].

In conclusion, our results show that the incidences of scrub typhus vary in different spatial settings, and the geographic distribution of scrub typhus appeared to have expanded over recent years, indicating the disease is emerging or re-emerging and remains an important public health problem in China. Meanwhile, the study also prove environmental factors such as temperature, precipitation and vegetation type are important drivers in the dynamics of scrub typhus. To the best of our knowledge, this is the most detailed study on spatiotemporal epidemiology of scrub typhus across the entire country, which provides a sound evidence base for future prevention and control programs and also lays a foundation for further investigation into the social and environmental factors responsible for changing disease patterns. Given the exponential growth and spatiotemporal features observed in this study, it is likely that the incidence of scrub typhus will increase in the future, and the disease may be spreading even to non-traditional foci where cases had rarely been reported. Moreover, based on the results of this study, it is recommended that immediate measures be taken in high-risk areas to increase health education and awareness of scrub typhus, enhance the availability of diagnostic and treatment practices, as well as continue surveillance of this emerging infectious disease.

## Supporting Information

S1 Checklist Strobe Checklist. (DOC)
