# Supplementary material for: Spatiotemporal Dynamics of Scrub Typhus Transmission in Mainland China, 2006-2014
Source: PLoS Negl Trop Dis. 2016 Aug 1;10(8):e0004875. doi: 10.1371/journal.pntd.0004875 (PMC4968795; doi:10.1371/journal.pntd.0004875)
Supplement: S1 Checklist — (DOC) [file pntd.0004875.s001.doc]

STROBE Statement—Checklist of items that should be included in reports of ***cross-sectional studies***

|  | Item No | Recommendation |
| --- | --- | --- |
| **Title and abstract** | 1 | (*a*) Indicate the study’s design with a commonly used term in the title or the abstract  **Cross-sectional design is listed in the revised abstract** |
|  |  | (*b*) Provide in the abstract an informative and balanced summary of what was done and what was found  **We explored the spatiotemporal dynamics of scrub typhus cases in China between January 2006 and December 2014, and explored the potential risk factors affecting the spatial distribution of the disease. The majority of cases were reported between July and November, with peak incidence during October every year. Several high-risk clusters were identified in southwest, southern, and middle-east China. Scrub typhus incidence was positively correlated with the percentage of shrub, and temporal variation in temperature and precipitation in China.** |
| Introduction | | |
| Background/rationale | 2 | Explain the scientific background and rationale for the investigation being reported  **Page 5 of revised cleaned version, lines 1-21; Page 6, lines 1-11.** |
| Objectives | 3 | State specific objectives, including any prespecified hypotheses  **Page 6 of revised cleaned version, lines 13-18 lists the objective** |
| Methods | | |
| Study design | 4 | Present key elements of study design early in the paper **N/A** |
| Setting | 5 | Describe the setting, locations, and relevant dates, including periods of recruitment, exposure, follow-up, and data collection  **Page 7 of revised cleaned version, lines 9-21 and page 8, lines 1-7.** |
| Participants | 6 | (*a*) Give the eligibility criteria, and the sources and methods of selection of participants  **Page 7 of revised cleaned version, lines 9-11.** |
| Variables | 7 | Clearly define all outcomes, exposures, predictors, potential confounders, and effect modifiers. Give diagnostic criteria, if applicable  **Page 8 of revised cleaned version, lines 1-7.** |
| Data sources/ measurement | 8* | For each variable of interest, give sources of data and details of methods of assessment (measurement). Describe comparability of assessment methods if there is more than one group  **Page 8 of revised cleaned version, lines 1-7.** |
| Bias | 9 | Describe any efforts to address potential sources of bias  **Data from January 2006 through December 2014 were obtained from** **China Information System for Disease Control and Prevention. All scrub typhus cases were confirmed according to the diagnostic criteria issued by the Ministry of Health of the People’s Republic of China and only clinical and laboratory confirmed were included in this study.** |
| Study size | 10 | Explain how the study size was arrived at  **Data from January 2006 through December 2014 in mainland China were collected and the data set of cases was aggregated at the county level as the spatial unit for analysis.** |
| Quantitative variables | 11 | Explain how quantitative variables were handled in the analyses. If applicable, describe which groupings were chosen and why  **Study was primarily descriptive epidemiology.** |
| Statistical methods | 12 | (*a*) Describe all statistical methods, including those used to control for confounding  **Page 8 of revised cleaned version, lines 14-21; page 9 and page 10.** |
|  |  | (*b*) Describe any methods used to examine subgroups and interactions **N/A** |
|  |  | (*c*) Explain how missing data were addressed **N/A** |
|  |  | (*d*) If applicable, describe analytical methods taking account of sampling strategy **N/A** |
|  |  | (*e*) Describe any sensitivity analyses **N/A** |
| Results | | |
| Participants | 13* | (a) Report numbers of individuals at each stage of study—eg numbers potentially eligible, examined for eligibility, confirmed eligible, included in the study, completing follow-up, and analysed  **page 11 of revised cleaned version, lines 4-5.** |
|  |  | (b) Give reasons for non-participation at each stage **N/A** |
|  |  | (c) Consider use of a flow diagram **N/A** |
| Descriptive data | 14* | (a) Give characteristics of study participants (eg demographic, clinical, social) and information on exposures and potential confounders  **page 11 of revised cleaned version, lines 4-5.** |
|  |  | (b) Indicate number of participants with missing data for each variable of interest **N/A** |
| Outcome data | 15* | Report numbers of outcome events or summary measures **N/A**  **page 14 of revised cleaned version, lines 8-20.** |
| Main results | 16 | (*a*) Give unadjusted estimates and, if applicable, confounder-adjusted estimates and their precision (eg, 95% confidence interval). Make clear which confounders were adjusted for and why they were included **N/A** |
|  |  | (*b*) Report category boundaries when continuous variables were categorized **N/A** |
|  |  | (*c*) If relevant, consider translating estimates of relative risk into absolute risk for a meaningful time period **N/A** |
| Other analyses | 17 | Report other analyses done—eg analyses of subgroups and interactions, and sensitivity analyses **N/A** |
| Discussion | | |
| Key results | 18 | Summarise key results with reference to study objectives  **page 15 of revised cleaned version, lines 1-9.** |
| Limitations | 19 | Discuss limitations of the study, taking into account sources of potential bias or imprecision. Discuss both direction and magnitude of any potential bias  **page 17 of revised cleaned version, lines 12-21.** |
| Interpretation | 20 | Give a cautious overall interpretation of results considering objectives, limitations, multiplicity of analyses, results from similar studies, and other relevant evidence  **page 18 of revised cleaned version, line 1-17.** |
| Generalisability | 21 | Discuss the generalisability (external validity) of the study results  **N/A** |
| Other information | | |
| Funding | 22 | Give the source of funding and the role of the funders for the present study and, if applicable, for the original study on which the present article is based  **YES** |

*Give information separately for exposed and unexposed groups.

**Note:** An Explanation and Elaboration article discusses each checklist item and gives methodological background and published examples of transparent reporting. The STROBE checklist is best used in conjunction with this article (freely available on the Web sites of PLoS Medicine at http://www.plosmedicine.org/, Annals of Internal Medicine at http://www.annals.org/, and Epidemiology at http://www.epidem.com/). Information on the STROBE Initiative is available at www.strobe-statement.org.
